# Phytogenic Synthesis of Pd-Ag/rGO Nanostructures Using Stevia Leaf Extract for Photocatalytic H_2_ Production and Antibacterial Studies

**DOI:** 10.3390/biom11020190

**Published:** 2021-01-29

**Authors:** Koduru Mallikarjuna, Omaima Nasif, Sulaiman Ali Alharbi, Suresh V. Chinni, Lebaka Veeranjaneya Reddy, Minnam Reddy Vasudeva Reddy, Subramaniam Sreeramanan

**Affiliations:** 1 Department for Management of Science and Technology Development, Ton Duc Thang University, Ho Chi Minh City 758307, Vietnam; koduru.mallikarjuna@tdtu.edu.vn; 2 Faculty of Applied Sciences, Ton Duc Thang University, Ho Chi Minh City 758307, Vietnam; 3 Department of Physiology, College of Medicine, King Saud University, Medical City, King Khalid University Hospital, P.O. Box 2925, Riyadh 11461, Saudi Arabia; onasif@ksu.edu.sa; 4 Department of Botany and Microbiology, College of Science, King Saud University, P.O. Box 22452, Riyadh 11495, Saudi Arabia; sharbi@ksu.edu.sa; 5 Department of Biotechnology, Faculty of Applied Sciences, AIMST University, Bedong 08100, Malaysia; v_suresh@aimst.edu.my; 6 Department of Microbiology, Yogi Vemana University, Kadapa, Andhra Pradesh 516005, India; 7 School of Chemical Engineering, Yeungnam University, 280 Daehak-ro, Gyeongsan-si 38541, Gyeongsangbuk-do, Korea; 8 School of Biological Sciences, Universiti Sains Malaysia (USM), Minden Heights 11800, Penang, Malaysia; 9 School of Bioprocess Engineering, Universiti Malaysia Perlis (UNIMAP), 02600 Arau, Perlis, Malaysia; 10 National Poison Centre, Universiti Sains Malaysia (USM), Minden Heights 11800 Penang Malaysia

**Keywords:** stevia extract, green synthesis, Pd-Ag/rGO, H_2_ production, antimicrobial activity, photocatalysis

## Abstract

Continuously increasing energy demand and growing concern about energy resources has attracted much research in the field of clean and sustainable energy sources. In this context, zero-emission fuels are required for energy production to reduce the usage of fossil fuel resources. Here, we present the synthesis of Pd-Ag-decorated reduced graphene oxide (rGO) nanostructures using a green chemical approach with stevia extract for hydrogen production and antibacterial studies under light irradiation. Moreover, bimetallic nanostructures are potentially lime lighted due to their synergetic effect in both scientific and technical aspects. Structural characteristics such as crystal structure and morphological features of the synthesized nanostructures were analyzed using X-ray diffraction and transmission electron microscopy. Analysis of elemental composition and oxidation states was carried out by X-ray photoelectron spectroscopy. Optical characteristics of the biosynthesized nanostructures were obtained by UV-Vis absorption spectroscopy, and Fourier transform infrared spectroscopy was used to investigate possible functional groups that act as reducing and capping agents. The antimicrobial activity of the biosynthesized Pd-Ag-decorated rGO nanostructures was excellent, inactivating 96% of *Escherichia coli* cells during experiments over 150 min under visible light irradiation. Hence, these biosynthesized Pd-Ag-decorated rGO nanostructures can be utilized for alternative nanomaterial-based drug development in the future.

## 1. Introduction

In recent years, the world has witnessed rapid industrial growth; energy demand and environmental pollution have been major issues. The majority of global energy demand is fulfilled by nonrenewable energy resources, the consumption of which is increasing. Since there are limited fossil fuel resources, there is significant concern about future demand for this finite energy source. As an alternative, hydrogen fuel is considered to be a sustainable and renewable energy source with zero carbon emissions that could help resolve energy demands and environmental concerns. Currently, the generation of hydrogen from solar-driven water splitting using semiconductor photocatalysts and photoelectrochemical cells (PECs) has attracted significant attention owing to their high production rate [1,2]. Reduced graphene (rGO) is an excellent candidate for the manufacture of new complexes, containing sp^2^-hybridized carbon atoms with a two-dimensional (2D) structure, and has generated keen interest from researchers because of its specific characteristics such as high conductivity, large surface area, and stability in different chemical environments. In rGO-based composites, generated photoelectrons can transfer quickly across the energy bands owing to the high work function of the graphene, which encourages the reactivity of adjacent materials [3,4,5,6,7]. Various materials have been used as photocatalysts for hydrogen production by the light-harvesting method. However, the activity of single-phase photocatalysts is limited by their bandgap and carrier recombination rate. To enhance the photocatalytic activity of semiconductors toward the visible region, researchers have dedicated efforts to tuning the bandgap of semiconductors by doping, modification of shape and surface, and combination with other semiconductors. Noble nanomaterials are used to generate the plasmonic effect on the surface of semiconductors, which can enhance photocatalytic activity [8,9]. Metal nanoparticles have attracted attention because of their exceptional physicochemical characteristics for the generation of hydrogen through the surface plasmon resonance (SPR) effect. This effect occurs when the frequency of the incident light matches the band gap between oscillating electrons and positive nuclei. Moreover, if the metal nanomaterial is optically active, it acts as a photosensitizer that can enhance photocatalytic activity due to the SPR [10,11,12,13,14,15].

Among noble metals, palladium nanostructures have excellent electrochemical activity in graphene-based composites and adsorbent materials [16]. However, the commercial use of palladium is restricted by its high cost. This has led to an interest in enhancing the development of bimetallic nanostructures. Such nanostructures can exhibit synergistic properties when compared with the properties of individual metallic nanomaterials. The combination of palladium with other noble metals can create bimetallic nanostructures, and desirable characteristics can be obtained by controlling the composition of the metals therein. The combination of palladium and silver is an interesting possibility since the synergistic combination of palladium and silver (Pd-Ag) with rGO results in excellent electrochemical activity [17,18,19].

Recently, bimetallic nanoparticles have attracted considerable attention because of their fascinating physical and chemical properties. Owing to the threat posed by multidrug-resistant pathogens, demand for the development of novel and efficient antimicrobial agents is increasing. Silver nanoparticles (Ag NPs) are well known for their antimicrobial activity through membrane damage and oxidation of cellular materials [19]. Graphene-based materials, graphene oxide (GO) and reduced GO (rGO), have been studied for their antimicrobial potential. Tan et al. [20] reported the synergistic and enhanced antimicrobial effect of GO/AgNPs on *Escherichia coli* and multidrug-resistant *Klebsiella pneumoniae*. Graphene-based nanomaterials are very attractive because of their biocompatibility; hence, they are used in biosensing, bioimaging, and as drug-delivery agents [21]. In particular, the photocatalytic effect of Ag and graphene is an inherent characteristic and can be used to target therapies with controllable light irradiation. Manikandan et al. [22] studied the biosynthesis of palladium nanoparticles and their antimicrobial properties using *Prunus yedoensis* leaf extract. Surendra et al. 2016 optimized Moringa oleifera peel extract for green synthesis of M. oleifera capped palladium nanoparticles using response surface methodology (RSM) and evaluated their antibacterial and hemolytic properties [23].

Considering the above, the development of new photocatalysts and eco-friendly methods using plants and microorganisms is of great interest owing to their cost-effectiveness, non-toxicity, and ease of production at a commercial scale. In the case of microbial synthesis, there is a complicated protocol for microbial screening and cultivation. In contrast, plant-based biosynthesis is more advantageous because of non-protocol mechanisms, low toxicity, and economic availability [24,25]. Quantitative phytochemical tests of plants have revealed the major and minor components, such as proteins, carbohydrates, peptides, saponins, and tannins. Moreover, these components can be used to reduce and stabilize synthesized nanoparticles [26,27].

Herein, we fabricated a Pd-Ag/rGO nanocomposite material using a green synthesis method by using stevia extract, studied its photocatalytic effect on hydrogen production, and evaluated its antimicrobial activity. This work can provide simple steps for the synthesis of nanomaterials by inexpensive and high-efficiency photocatalysis.

## 2. Materials and Methods

Palladium chloride (PdCl_2_), silver nitrate (AgNO_3_), potassium permanganate (KMnO_4_), and graphite flakes were procured from Sigma-Aldrich (St. Louis, MO, USA). Plant leaf broth of stevia was prepared from 5 g of stevia leaves placed in a 250 mL conical flask with 100 mL of DI water. The mixture was boiled to obtain the extract, then cooled to room temperature and filtered with Whatman no. 1 filter paper (pore size 20 μm). The stevia extract is a rich source of amides, proteins, polyphenols, and flavonoids.

### 2.1. Preparation of Graphene Oxide (GO)

To prepare GO using a modified Hummer method, 2 g of graphite flakes and sodium nitrite were added to concentrated H_2_SO_4_ in a three-neck round-bottom flask with continuous stirring at low temperature using an ice bath. Next, 12 g of KMnO_4_ was slowly added to the reaction mixture which was then refluxed for 15 min. Subsequently, 40 mL of H_2_O_2_ followed by 200 mL of distilled water were added to the reaction mixture, and stirring was continued for 4 h. The obtained mixture was washed several times with ethanol and water, and the final compound was vacuum dried at 60 °C for 8 h.

### 2.2. Preparation of Pd-Ag/Reduced Graphene Oxide (rGO) and Pd/rGO

In the case of the Pd-Ag/rGO nanostructures, 1 g of prepared GO, 50 mg of PdCl_2,_ and 50 mg of AgNO_3_ were added to 200 mL of DI water and stirred for 1 h to disperse the GO. Stevia extract (25 mL) was added to the mixture and stirring was continued for 3 h. Similarly, for the Pd/rGO nanostructures, 1 g of GO and 100 mg of PdCl_2_ were added to 200 mL of DI water and stirring was continued for 1 h to prepare a homogeneous mixture. Then, 25 mL of extract was added to the above solution and stirring was continued for 3 h.

### 2.3. Characterization

The phase purity of the prepared materials was studied by X-ray diffraction (XRD, PANalytical X’Pert PRO, Malvern, UK) with high-intensity Cu Kα_1_ (λ = 1.541 Å) radiation. The microscopic features of size, shape, and distribution of the prepared structures were analyzed using transmission electron microscopy (TEM; FEI Tecnai G2 F20 Twin, Hillsboro, OR, USA) with an operating voltage of 200 kV. The optical absorption properties of the prepared materials were determined using a UV-visible absorption spectrophotometer (UV-Vis; Genesys 10S, Marietta, OH, USA) with a resolution of 1 nm. The functional groups of biomolecules, which act as reducing and capping agents for palladium-silver/rGO nanoparticles, were studied using Fourier transform infrared (FTIR; Thermo Scientific, Waltham, MA, USA) spectroscopy with a resolution of 4 cm^−1^.

### 2.4. Photocatalytic Hydrogen Evaluation

Photocatalytic hydrogen production from water splitting under an artificial solar simulator (light intensity of ~120,000 Lux) was studied using methanol as a scavenger. To eliminate dissolved oxygen, the reactor vessel was purged with nitrogen before light illumination. After illumination, the generated hydrogen gas was collected and analyzed using a chromatograph (Chemito; TCD detector, 5 Å molecular sieve column).

### 2.5. Antimicrobial Activity of Pd/Ag/rGO

The *Escherichia coli* K-12 was selected as a model test organism for photocatalytic inactivation tests. The microorganism was propagated in 50 mL of LB broth (Difco, France) incubated at 150 rpm and 37 °C for 12 h to obtain 10^6^ cells/mL. The propagated microbial cells were pelleted and cell debris was removed by washing the pellets twice with sterilized saline solution (0.9% NaCl) and dissolved in saline solution with a final cell number of about 1 × 10^6^ CFU/mL. Pre-dispersed Pd/Ag/rGO NP samples were added to the bacterial suspension at a final concentration of 1 mg/mL. Finally, the solution was transferred to a 10 mL jacketed round-bottom flask and placed in a light system fabricated in our laboratory and subjected to one sunlight for photoactivation. The agar plate preparation and method of viable bacterial count were carried out as described by Lingamdinne et al. [28].

## 3. Results

### 3.1. UV-Visible Analysis

**Figure 1:**
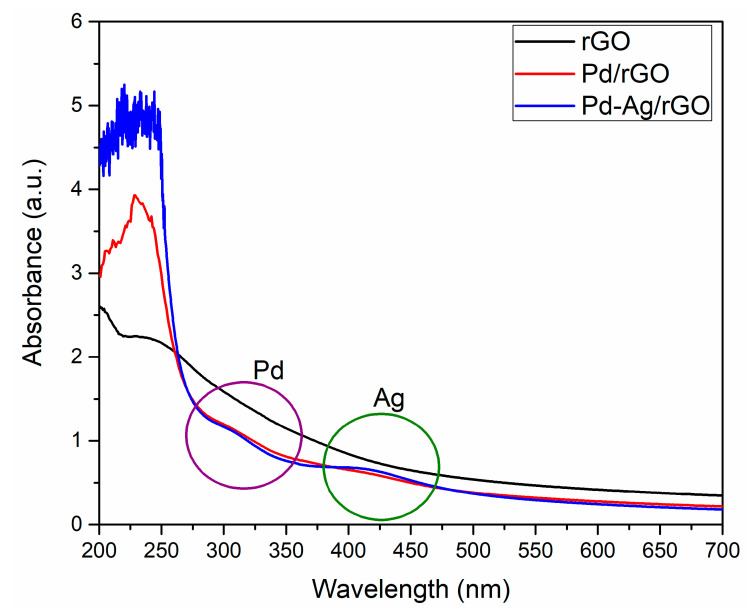
UV-Vis absorption spectra of prepared rGO, Pd/rGO, and Pd-Ag/rGO nanostructures using plant leaf extract.

The formation of nanoparticles was confirmed by measuring the optical absorption spectra of rGO, Pd/rGO, and Pd-Ag/rGO, and the results are shown in Figure 1. The obtained results confirm the formation of rGO with nanoparticles on the rGO nanostructure. Moreover, it is known that the interaction of light with conduction electrons in the conduction band causes the SPR effect. The size and morphology of nanoparticles play a vital role in producing changes in the intensity and position of the SPR peak. The SPR peak at ~320 nm indicates the formation of Pd nanoparticles [27]. In contrast, rGO nanosheets show absorption in the scanned wavelength range [29]. The formation of nano-junctions between rGO and Pd nanoparticles causes variation in the intensity of the absorption peak of Pd/rGO, and a similar effect was observed in Pd-Ag/rGO, where an SPR peak for Ag nanoparticles is observed in the range 380–450 nm [29]. However, the addition of Ag with Pd/rGO dominates the SPR peak of Ag nanoparticles and shifts the absorption from the UV to the visible range. The SPR peak of Ag nanoparticle is flattened and merges with the SPR peak of Pd nanoparticles.

### 3.2. XRD Analysis

**Figure 2:**
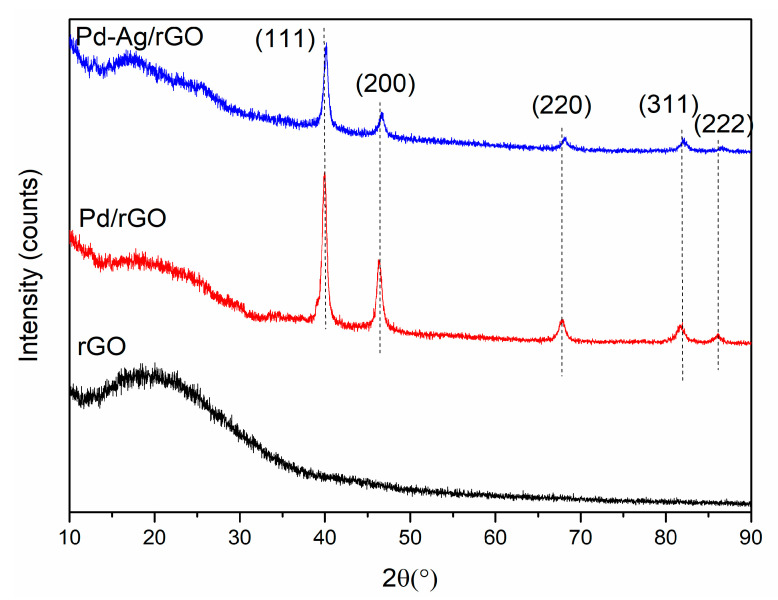
X-ray diffraction results of synthesized materials of rGO, Pd/rGO, and Pd-Ag/rGO nanostructures.

The crystalline signature of the prepared nanoparticles was determined by XRD analysis and the results are shown in Figure 2. It can be seen that the high-intensity peak of rGO disappears upon the addition of Pd and Pd-Ag nanoparticles and new diffraction peaks are formed. For the Pd/rGO nanoparticles, the peaks at 2θ = 40.2°, 46.6°, 67.7°, 82.4°, and 87.6° are attributed to the (111), (200), (220), (311), and (222) facets of the crystal planes, respectively, which match perfectly with the standard diffraction pattern for pristine Pd (JCPDS: 68–2867) with a face-centered cubic (FCC) structure. Similarly, in the case of Pd-Ag/rGO, the same sharp peaks are obtained with a slight red shift (0.24°), which may be due to the Ag. These values lie between those of pristine Pd (JCPDS: 68–2867) and pristine Ag (JCPDS: 68–2871) with an FCC structure [30,31]. The average sizes of Pd/rGO and Pd-Ag/rGO were calculated using Debye Scherrer’s equation for a crystal plane (111), and the estimated sizes are ~13.4 nm and ~11.6 nm, consistent with TEM results.

### 3.3. FTIR Analysis

**Figure 3:**
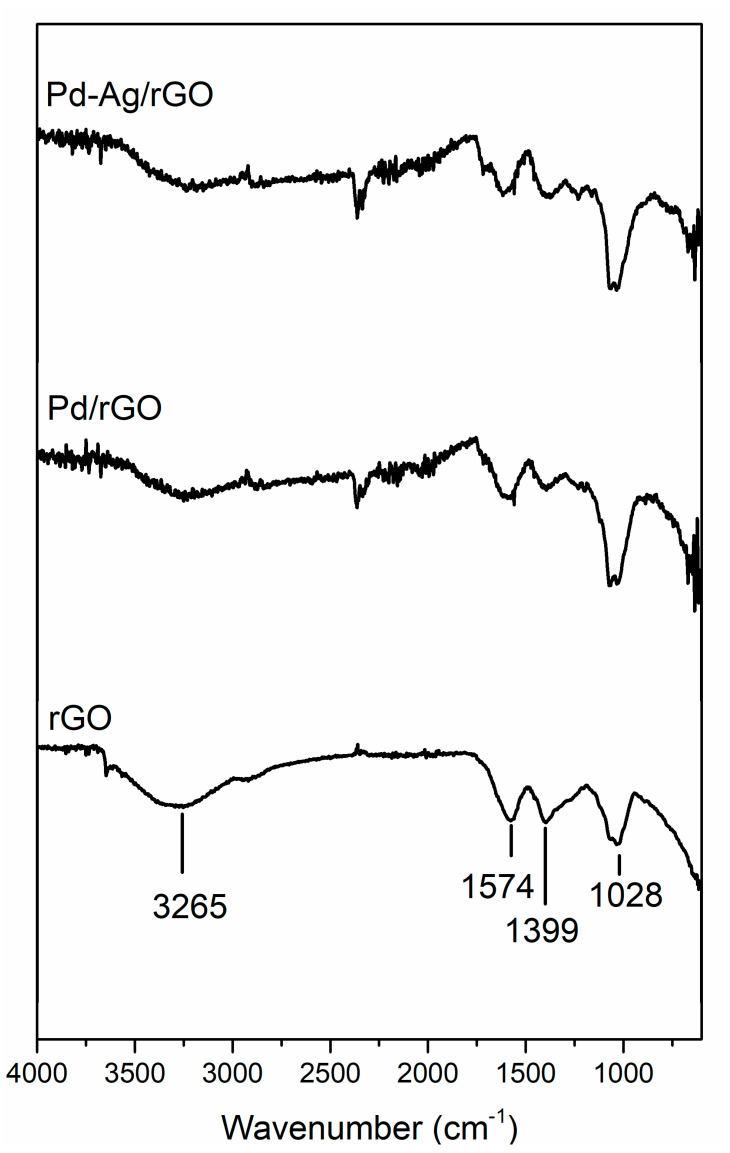
Fourier Transform Infrared (FTIR) spectra of fabricated GO, Pd/rGO, and Pd-Ag/rGO nanostructures using stevia leaf extract.

To identify the functional groups of the reducing and capping agents of nanoparticles with stevia leaf extract, FTIR analysis was carried out in the wavenumber range 4000–400 cm^−1^. In Figure 3, peaks are observed at 3265, 1574, 1399, and 1028 cm^−1^, and the band at 3265 cm^−1^ represents the C-N bending vibrations of amide groups of proteins in the presence of stevia extract. Moreover, the peak at 1574 cm^−1^ represents strong N-H stretching vibrations of the amide of proteins linked with surface materials. The peaks at 1399 and 1028 cm^−1^ correspond to strong C-O vibrations of alcohols and phenols and medium-to-strong C-N vibrations of amines and amides, respectively [28]. A peak similar to that was found at 1574 cm^−1^ in the rGO was observed with a blue shift in Pd/rGO at 1625 cm^−1^, and a corresponding shift was observed in Pd-Ag/rGO with a broader, less intense blue-shifted peak. The broader peak is due to GO surface functional groups C=O, C=C, and C-O, as confirmed by the presence of their respective bands at ~1743 cm^−1^, ~1629 cm^−1^, and ~1093 cm^−1^ [29]. This is evidence of the stevia extract acting as a reducing and capping agent of the Pd/rGO and Pd-Ag/rGO structures. From FTIR analysis, a possible pathway for the formation of nanoparticles and attachment of the nanoparticles on the rGO sheets may be because the carbonyl group in proteins have a stronger ability to bind with nanoparticles on the rGO surface. Furthermore, the surface layer of proteins probably covers the nanoparticles and prevents their agglomeration.

### 3.4. TEM Analysis

**Figure 4:**
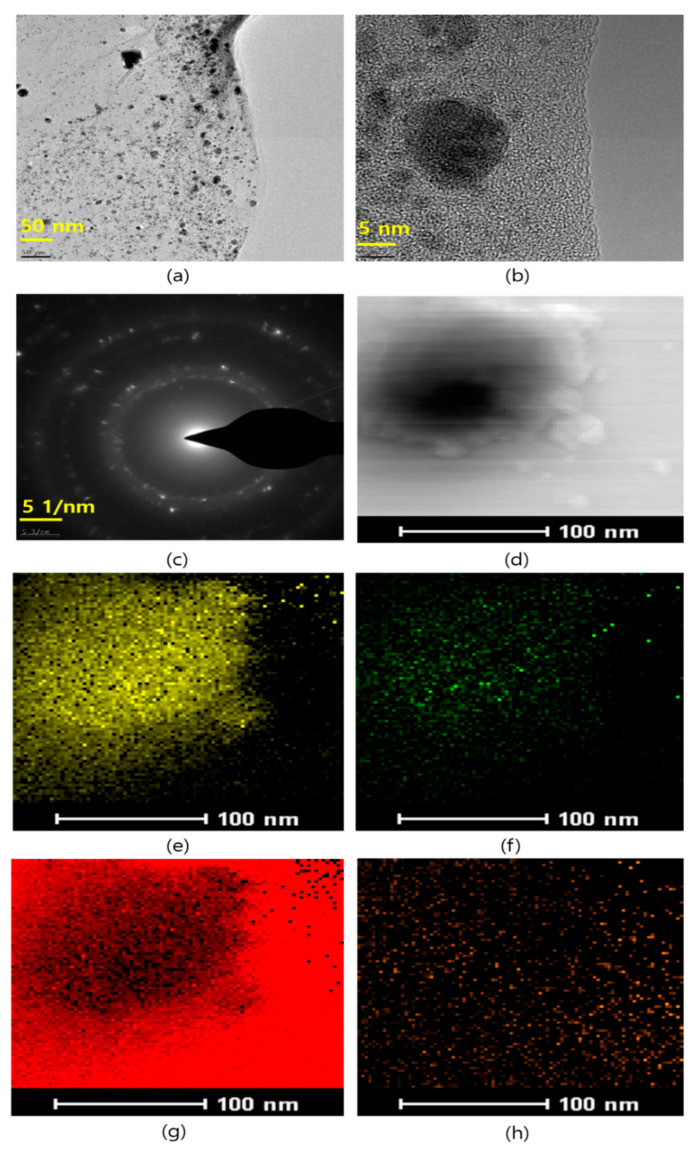
Different magnification TEM images of Pd-Ag nanoparticles anchored on reduced graphene oxide sheets (**a**) 50 nm (**b**) 5 nm (**c**) Selected Area Electron Diffraction (SAED) pattern (**d**) STEM-HAADF image and elemental mapping of (**e**) palladium (**f**) silver, (**g**) carbon and (**h**) oxygen elements presence in Pd-Ag/RGO nanostructures.

**Figure 5:**
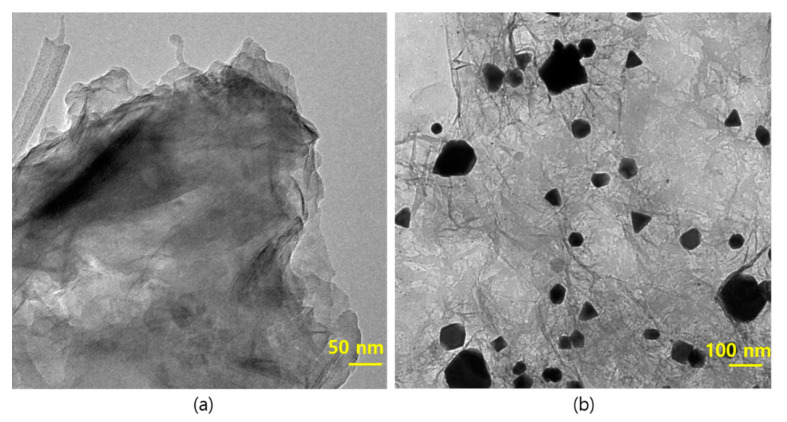
TEM images of Pd/rGO and rGO nanostructures. (**a**) rGO nanosheets, and (**b**) Pd nanostructures anchored on rGO nanosheets.

The morphological features of the prepared Pd-Ag/rGO were studied by TEM analysis and the results are shown in Figure 4. The TEM image revealed that the Pd-Ag nanoparticles are distributed on the rGO sheets (Figure 4a). The particles are spherical with a size of 5–15 nm, and few of the particles are agglomerated (Figure 4b). Furthermore, the low-dimensional particles are likely to be reactive owing to their high surface-to-volume ratio and active sites at the edges of the particles. When the size of particles is smaller, their surface-to-volume ratio is enhanced, which impacts their catalytic, optical, and chemical reactions. The crystalline nature of the optimized sample observed in the selected area electron diffraction (SAED) pattern in Figure 4c results from the tiny crystals of Pd-Ag nanoparticles. The dark contrast in the TEM images comes from Pd and the light contrast is from the Ag [31]. The elemental mapping suggests an even distribution of elements over the whole rGO surface, as shown in Figure 4e–h. The morphological features of Pd/rGO and rGO are represented in Figure 5, where the rGO is seen to have a multilayered structure (Figure 5a), while Pd/rGO has Pd nanoparticles anchored on rGO nanosheets was depicted in Figure 5b.

### 3.5. XPS Analysis

**Figure 6:**
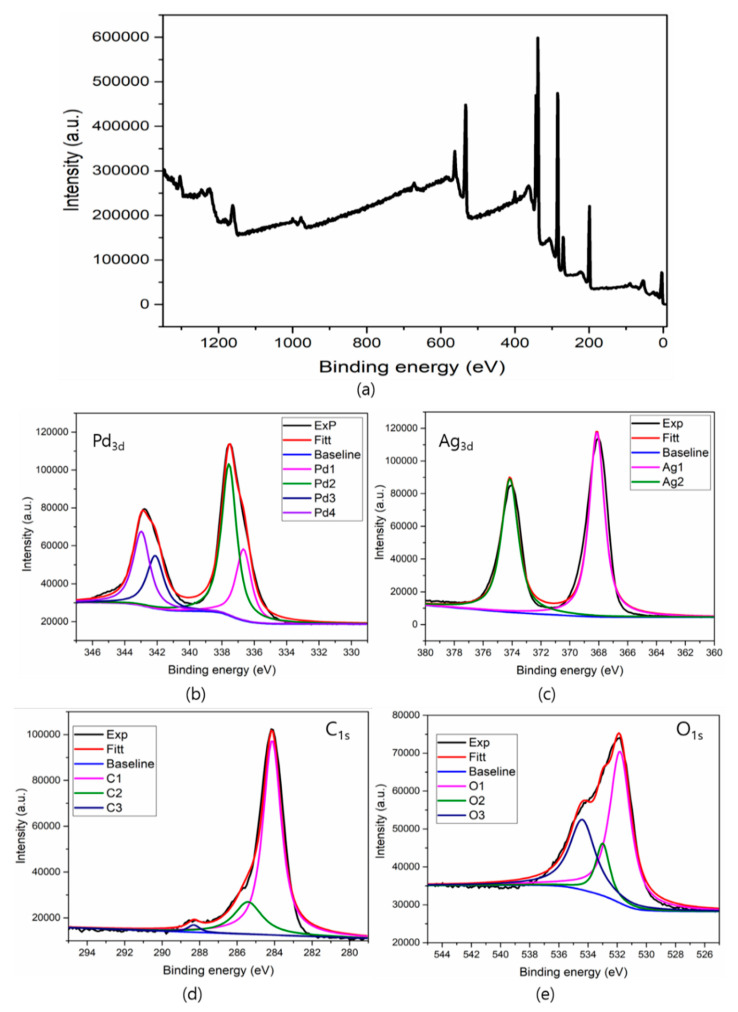
XPS spectra of Pd-Ag/RGO nanostructures (**a**) survey scan(**b**) Palladium (**c**) Silver (**d**) Crbon and (**e**) Oxygen.

The chemical components and oxidation states of the elements were investigated using XPS analysis. Figure 6a depicts the complete survey spectrum and confirms the elemental presence of Pd, Ag, C, Cl, N, and O in the synthesized composite. The high-resolution Pd spectrum (Figure 6b) shows strong double peaks at 342.9 and 337.6 7 eV with a difference of 5.2 eV, corresponding to Pd 3d_5/2_ and Pd 3d_3/2_, respectively. Figure 6c shows double peaks located at 368.1 eV and 374.1 eV with a difference of 6.0 eV, corresponding to Ag 3d_5/2_ and Ag 3d_3/2_ of metal silver. The shift in the lower binding energy may be due to the interaction of the Pd-Ag nanostructures with rGO. In Figure 6d the broad spectra of C 1s, with peaks at 284.1, 285.4, and 288.3 eV were observed, which can be attributed to the sp^2^ C-C/C=C, sp^3^ C-C, and O-C=O, correspondingly. Moreover, the high-resolution spectra of O 1s (Figure 6e) represents the peaks at 531.8, 533.0, and 536.4 eV attributed to the C=O, O-H and C-OOH bonds, respectively, attributed to the rGO [32]. The interaction of Pd-Ag nanostructures with rGO is attributed to the C=O group, because of the strong peaks for O 1s and C 1s in the spectra. The inherent nature of the C 1s peak of the nanocomposite contains many components of C-C and C=O, and the carbon content is derived from biomolecules [33]. It is strongly suggested that the carbonyl groups in proteins (which are present in leaf extract) have a strong ability to bind with nanoparticles on the rGO surface.

### 3.6. Hydrogen Production

**Figure 7:**
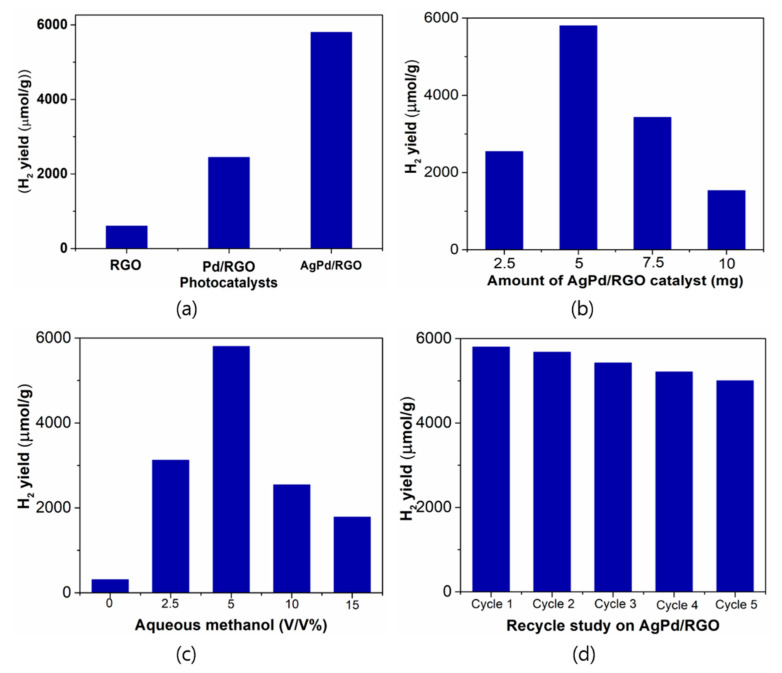
(**a**) Photocatalytic H_2_ production with prepared catalysts (**b**) Different amounts of Pd-Ag/rGO catalyst for H_2_ production (**c**) Different amounts of sacrificial agent (methanol) for H_2_ production (**d**) Reusability test for H_2_ production using Pd-Ag/rGO photocatalyst.

Figure 7 shows the photocatalytic activity of the rGO, Pd/rGO, and Pd-Ag/rGO samples for hydrogen production. The rGO sample produces very little hydrogen because of low light absorption by the sample. Moreover, the samples with presence of metal and bimetallic nanoparticles with rGO produces high H_2_ production, because of SPR. The hydrogen evolution is higher in Pd-Ag/rGO than in Pd/rGO (Figure 7a). The sample’s high surface area with low bandgap energy and low electron-hole recombination rate results in more photocatalytic activity for hydrogen production. Hence, Pd-Ag/rGO is a good photocatalyst for the evolution of hydrogen. The effect of increasing the dose of the Pd-Ag/rGO photocatalyst is shown in Figure 7b. The results show that hydrogen evolution is 2542, 5802, 3432, and 1534 µmol g^−1^ for photocatalyst doses of 2.5, 5, 7.5, and 10 mg, respectively, indicating that as the dose initially increases to the optimal range of 5 mg, the amount of hydrogen evolution increases, as more active site availability increases the rate of photocatalytic activity. Doses above 5 mg of photocatalyst decrease the hydrogen evolution because there is less penetration of light to reach the catalyst surface, and thus the photocatalytic activity is decreased [34]. Moreover, we investigated the optimization of sacrificial agent addition during water splitting using an optimized sample (Figure 7c). With an increasing methanol concentration up to 5%, the rate of H_2_ production increases, but above 5% methanol, the rate of H_2_ production decreases because of the generation of methanol byproducts in the converted polar medium. Determining the stability is one of the key parameters for usage catalysts in large scale applications. In the reusability test (Figure 7d), the H_2_ production decreases slightly with each cycle, suggesting that as the sample is reused, there is less interaction with the surface of the catalyst in the oxidation process, resulting in decreasing H_2_ production in successive cycles.

**Figure 8:**
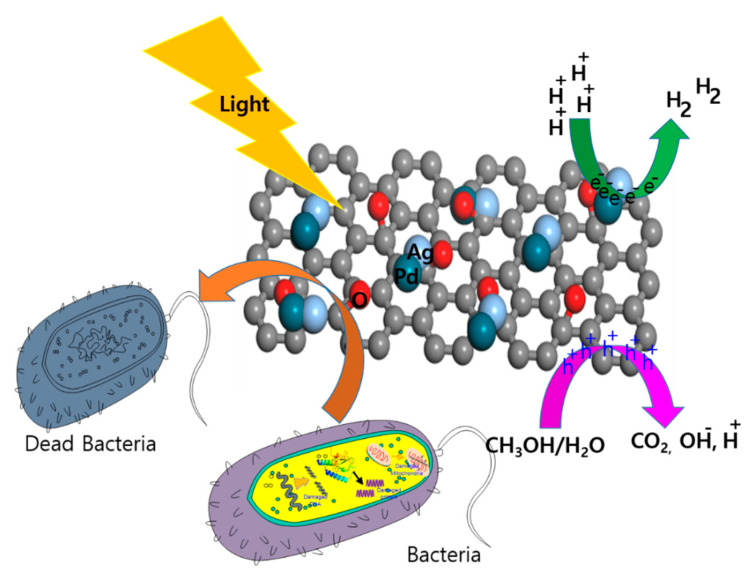
Schematic representation of photocatalytic H_2_ production and hypothetical antimicrobial mechanism of prepared catalysts.

Synergy exists between Pd and Ag and the two catalytic cycles work in concert to create a single new entity, i.e., Pd-Ag as the active phase. It can be seen by TEM that the Pd-Ag/rGO nanocomposite has the best contact surface and the best interaction forces, and therefore is most suitable as a visible light-activated catalyst for the production of H_2_ by photocatalytic water-splitting. Based on the experimental results, we suggest a pathway for the upgraded water-splitting reaction, as depicted in Figure 8. Under light irradiation, both Pd-Ag and rGO are excited, generating electrons and holes. Photoinduced charge carrier electrons are transported from the rGO to the Pd-Ag to react with H^+^, which in turn produces hydrogen. Meanwhile, the valence band (VB) holes of rGO react with the aqueous methanol solution. Then, the charge carriers participate in oxidation/reduction reactions in the methanol-aqueous reactor. When the Pd-Ag bimetal interacts with rGO, novel energy positions are generated at the edge to create supplementary active sites for water-splitting. However, the robust synergy between Pd and Ag appears to prevent the recombination of electrons and holes, possibly because of the high storage capacity of photoexcited electrons in Pd-Ag. For example, Mandari et al. reported that Ag-Cu/TiO_2_ catalyst facilitates electron-hole separation, and a solid interface between Ag and Cu led to metallic sites (Ag, Cu, and/or Ag-Cu) acting as electron traps [34,35]. According to J. Li et al., the loading of two precious metals promotes the photogeneration and separation of electrons and holes, while the synergy of Ag and Pd bimetals also inhibits the recombination of photoinduced electrons and holes [36,37,38,39]. Therefore, the superior photocatalytic performance of Pd-Ag/rGO is mainly because of Pd-Ag, which can act as an electron sink to inhibit the recombination rate and provide more catalytically active sites for efficient H_2_ evolution.

### 3.7. Antimicrobial Activity of Pd/Ag/rGO

**Figure 9:**
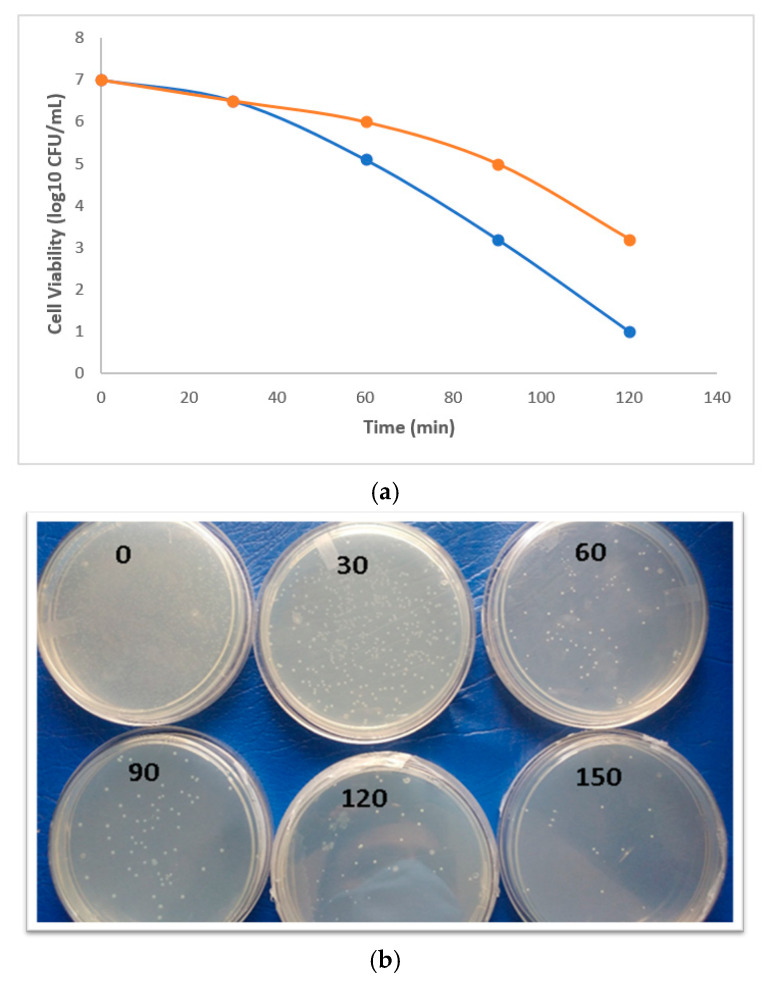
(**a**) Bacterial inhibition kinetics of Pd-Ag/rGO on *E.* *coli* K-12 in the presence of light (Blue line) and in the dark (Red line). (**b**) Bacterial inhibition performance of Pd-Ag/rGO on *E. coli* K-12 with light.

The photocatalytic microbial inhibition activity of the biosynthesized Pd/Ag/rGO NPs was tested with *Escherichia coli* K-12 with and without light. Pd/Ag/rGO inactivated or killed 96% of the bacteria under visible light irradiation within 150 min (2.5 h) (Figure 9a,b). Bacterial inactivation in dark experiments indicated an inhibition effect of 55%. The obtained results suggest that Pd/Ag/rGO NPs are more effective at killing the selected microbe in the presence of light than in dark conditions. The antimicrobial potential of Pd/Ag/rGO NPs is time-dependent and the inactivation of microbes increases with incubation time. The inactivation capability is clearly visible after 30 min of incubation. Approximately 75% of the inactivation appears after 90 min of incubation, suggesting very fast action (Figure 8 and Figure 9a,b). The antimicrobial mechanism of graphene involves both physical and electronic interactions. Physical interactions can include cell-wrapping, cell trapping, and membrane rupture by edges of the graphene. Electronic interaction can include contact with semi-metallic rGO electrons transferred from the cell membrane by Schottky barrier formation, damaging membrane integrity and creating chemical oxidative stress, which leads to the cessation of microbial growth through deactivation of amino acids that make up proteins, DNA, and cell membrane molecules by reactive oxygen species [21,22,23,27,28]. The antibacterial property of the Ag-rGO composites is thought to be because of the well-accepted mechanism of capturing and killing. Physical and chemical reactions can be a source of free radicals or reactive oxygen species. Microbial inactivation through photocatalysis is caused by photogenerated electrons and holes. Under light irradiation, Pd, Ag, and rGO produce electron-hole pairs, and some of them will reach the surface of Pd/Ag/rGO and cause the separation of electron-hole pairs. The conduction band (CB) of Pd/Ag/rGO is highly negative compared to that of the O_2_/O^2−^ potential, which leads to the reduction of O_2_ to O^2−^. Superoxide species generated in this way can also kill microbial cells by damaging the bacterial membrane and inactivating the genetic material by disrupting phosphate and hydrogen bonds. The rGO sheets with their large specific surface area can capture bacteria, increasing the opportunity for contact between the Ag and Pd NPs and bacteria that increases antimicrobial activity. The selected gram-negative *E. coli* potentially inhibited by Ag and Pd nanoparticles may be due to the easy entry into the cytoplasm through the thin outer membrane and rapid inhibition of metabolic activities. In addition, stevia leaf extract phytochemicals, which act as capping agents, may contribute to bacterial inactivation. These results are in good agreement with previous reports on rGO/Ag and Pd NPs [23,40,41]. In this photoexcitation, OH radicals and H_2_O_2_ species are also produced, which can play an important role in the inactivation of bacterial cells [4]. In addition to the above, Pd^2+^ is well known as an enzyme inhibitor and known to inhibit creatine kinase, succinate dehydrogenase, and many other very important enzymatic processes in both prokaryotic and eukaryotic cells [23,42,43].

## 4. Conclusions

We successfully synthesized Pd-Ag-decorated rGO nanostructures using a green chemical approach with stevia extract for hydrogen generation under light irradiation. TEM analysis revealed that the Pd-Ag nanoparticles were homogeneously dispersed on the rGO sheets, and the size of the particles was less than 10 nm with a spherical shape. The particle distribution and surface morphology of the prepared Pd-Ag nanoparticles on rGO sheets suggest less agglomeration. The Pd-Ag nanoparticles are spherical with even distribution on the rGO sheets. Moreover, the enhanced photocatalytic property for hydrogen production was due to a synergetic effect of Pd-alloy formation and SPR created by the silver. The antimicrobial activity of the biosynthesized Pd-Ag-decorated rGO nanostructures was excellent and inactivated 96% of *E. coli* cells during experiments lasting 150 min under visible light irradiation. The selected gram-negative *E. coli* inhibition by the Ag and Pd nanoparticles may be due to easy entry into the cytoplasm through the thin outer membrane and rapid inhibition of metabolic activities. Including the above stevia leaf extract, phytochemicals, which act as capping agents, may contribute to bacterial inactivation. It can be concluded that the reported green synthesis procedure presents functionalized 2D materials and biosynthesized Pd-Ag-decorated rGO nanostructures that are favorable for energy storage and environmental applications and can be used for alternative nanomaterial-based drug development in the future.

## Data Availability

Data available on request due to restrictions.
